# Supplementary material for: Partial response electromyography as a marker of action stopping
Source: eLife. 2022 May 26;11:e70332. doi: 10.7554/eLife.70332 (PMC9203056; doi:10.7554/eLife.70332)
Supplement: Transparent reporting form [file elife-70332-transrepform1.pdf]

eLife's transparent reporting form

We encourage authors to provide detailed information *within their submission* to facilitate the interpretation and replication of experiments. Authors can upload supporting documentation to indicate the use of appropriate reporting guidelines for health-related research (see EQUATOR Network), life science research (see the BioSharing Information Resource), or the ARRIVE guidelines for reporting work involving animal research. Where applicable, authors should refer to any relevant reporting standards documents in this form.

If you have any questions, please consult our Journal Policies and/or contact us: editorial@elifesciences.org.

Sample-size estimation

- You should state whether an appropriate sample size was computed when the study was being designed
- You should state the statistical method of sample size computation and any required assumptions
- If no explicit power analysis was used, you should describe how you decided what sample (replicate) size (number) to use

Please outline where this information can be found within the submission (e.g., sections or figure legends), or explain why this information doesn't apply to your submission:

The data was collected as a part of a larger study that investigates the behavioral, neural and muscular signatures of inhibitory control, and whether these signatures are stable over time. The analysis for this manuscript was performed after n reached 50, because this is considerably larger than the samples in the previous studies using prEMG, and offered a balanced trade-off between high expected power and time spent on data collection. No explicit power analysis was performed, since the planned replication effects (e.g. the comparison of the prEMG and the SSRT) have been very large in previous studies (Cohen's d > 2, significant effects reported with n = 10), effect size estimates for novel analyzes were lacking, and the range of analyzes could not be covered by a single power estimate.

Replicates

- You should report how often each experiment was performed
- You should include a definition of biological versus technical replication
- The data obtained should be provided and sufficient information should be provided to indicate the number of independent biological and/or technical replicates
- If you encountered any outliers, you should describe how these were handled
- Criteria for exclusion/inclusion of data should be clearly stated
- High-throughput sequence data should be uploaded before submission, with a private link for reviewers provided (these are available from both GEO and ArrayExpress)

Please outline where this information can be found within the submission (e.g., sections or figure legends), or explain why this information doesn't apply to your submission:

The data reported in this manuscript is the first experimental session in a larger study. Inclusion criteria included healthy adults between age 18-40 with no current or previous neurological or psychiatric disorders. Data was discarded due to technical issues during acquisition (n=4) and as statistical outliers (n=2). Sample information is found on manuscript p.27.

Statistical reporting

- Statistical analysis methods should be described and justified
- Raw data should be presented in figures whenever informative to do so (typically when N per group is less than 10)
- For each experiment, you should identify the statistical tests used, exact values of N, definitions of center, methods of multiple test correction, and dispersion and precision measures (e.g., mean, median, SD, SEM, confidence intervals; and, for the major substantive results, a measure of effect size (e.g., Pearson's r, Cohen's d)
- Report exact p-values wherever possible alongside the summary statistics and 95% confidence intervals. These should be reported for all key questions and not only when the p-value is less than 0.05.

Please outline where this information can be found within the submission (e.g., sections or figure legends), or explain why this information doesn't apply to your submission:

Detailed information about statistical tests and used software are presented in a corresponding paragraph in 'Materials and methods' section on manuscript p.29-31.

(For large datasets, or papers with a very large number of statistical tests, you may upload a single table file with tests, Ns, etc., with reference to sections in the manuscript.)

Group allocation

- Indicate how samples were allocated into experimental groups (in the case of clinical studies, please specify allocation to treatment method); if randomization was used, please also state if restricted randomization was applied
- Indicate if masking was used during group allocation, data collection and/or data analysis

Please outline where this information can be found within the submission (e.g., sections or figure legends), or explain why this information doesn't apply to your submission:

All comparative analyses were done on repeated measures data, thus there was no group allocation.

Additional data files ("source data")

- We encourage you to upload relevant additional data files, such as numerical data that are represented as a graph in a figure, or as a summary table
- Where provided, these should be in the most useful format, and they can be uploaded as "Source data" files linked to a main figure or table
- Include model definition files including the full list of parameters used
- Include code used for data analysis (e.g., R, MatLab)

eLife Sciences Publications, Ltd is a limited liability non-profit non-stock corporation incorporated in the State of Delaware, USA, with company number 5030732, and is registered in the UK with company number FC030576 and branch number BR015634 at the address 1st Floor, 24 Hills Road, Cambridge CB2 1JP | August 2014

- Avoid stating that data files are "available upon request"

Please indicate the figures or tables for which source data files have been provided:

The raw and processed data with all analysis scripts (Matlab, R) are uploaded publicly in the Open Science Framework.

Direct link: https://osf.io/RQNUJ/

Citation:
Raud L, Thunberg C, Huster R. 2021. Partial response electromyography as a marker of the individual stopping latency. Data and analyses scripts. doi:10.17605/OSF.IO/RQNUJ
